# β_2_-AR inhibition enhances EGFR antibody efficacy hampering the oxidative stress response machinery

**DOI:** 10.1038/s41419-023-06129-9

**Published:** 2023-09-19

**Authors:** Vitale Del Vecchio, Luigi Mele, Sameer Kumar Panda, Ibone Rubio Sanchez-Pajares, Laura Mosca, Virginia Tirino, Massimiliano Barbieri, Francesca Bruzzese, Antonio Luciano, Federica Zito Marino, Marina Accardo, Giovanni Francesco Nicoletti, Gianpaolo Papaccio, Antonio Barbieri, Vincenzo Desiderio

**Affiliations:** 1 Department of Experimental Medicine, University of Campania “Luigi Vanvitelli”, Naples, Italy; 2 Department of Precision Medicine, University of Campania “Luigi Vanvitelli”, Naples, Italy; 3 S.S.D. Sperimentazione Animale, Istituto Nazionale Tumori—IRCCS—Fondazione G. Pascale, Naples, Italy; 4 Department of Mental and Physical Health and Preventive Medicine, University of Campania “Luigi Vanvitelli”, Naples, Italy; 5 Multidisciplinary Department of Medical-Surgical and Dental Specialties, University of Campania “L. Vanvitelli”, Via L. de Crecchio 6, 80138 Naples, Italy

**Keywords:** Oral cancer, Mechanisms of disease

## Abstract

The β2-Adrenergic receptor (β2-ARs) is a cell membrane-spanning G protein-coupled receptors (GPCRs) physiologically involved in stress-related response. In many cancers, the β2-ARs signaling drives the tumor development and transformation, also promoting the resistance to the treatments. In HNSCC cell lines, the β2-AR selective inhibition synergistically amplifies the cytotoxic effect of the MEK 1/2 by affecting the p38/NF-kB oncogenic pathway and contemporary reducing the NRF-2 mediated antioxidant cell response. In this study, we aimed to validate the anti-tumor effect of β2-AR blockade and the synergism with MEK/ERK and EGFR pathway inhibition in a pre-clinical orthotopic mouse model of HNSCC. Interestingly, we found a strong β2-ARs expression in the tumors that were significantly reduced after prolonged treatment with β2-Ars inhibitor (ICI) and EGFR mAb Cetuximab (CTX) in combination. The β2-ARs down-regulation correlated in mice with a significant tumor growth delay, together with the MAPK signaling switch-off caused by the blockade of the MEK/ERK phosphorylation. We also demonstrated that the administration of ICI and CTX in combination unbalanced the cell ROS homeostasis by blocking the NRF-2 nuclear translocation with the relative down-regulation of the antioxidant enzyme expression. Our findings highlighted for the first time, in a pre-clinical in vivo model, the efficacy of the β2-ARs inhibition in the treatment of the HNSCC, remarkably in combination with CTX, which is the standard of care for unresectable HNSCC.

## Introduction

Beta-adrenergic receptors (β-ARs) are a family of proteins widely expressed in physiological as well as pathological conditions. Catecholamine epinephrine and norepinephrine are the biological agonizts of β-ARs that modulate the sympathetic nervous system (SNS)–induced fight-or-flight stress responses [1]. Specifically, β2-AR signaling is able to modulate metabolic pathways either in normal or pathological conditions. In fact, the β2-adrenergic receptor regulates ER–mitochondria contacts, being a regulatory pathway for ER–Mito coupling, and these contacts respond to physiological demands or stresses [2, 3]. On the other hand, Beta-adrenergic receptor gene polymorphisms are associated with cardiac contractility and blood pressure variability [4]. The key role of the β2-AR signaling in cancer biology has been initially demonstrated in epidemiological studies that correlated chronic stress with accelerated tumor progression [5, 6], as well as reduced tumor aggressiveness in patients under β-blockers therapy [7, 8]. The β2-AR signaling promotes tumor initiation and progression by regulating several cell processes, such as apoptosis/anoikis [9, 10], autophagy, angiogenesis [11], inflammation and immune-response [12, 13], DNA damage, drug resistance and EMT [14, 15].

In breast cancer, the β2-AR catecholamine activation negatively correlates with drug response in HER2 overexpressing patients, showing a PI3K/Akt/mTOR mediated resistance to the therapy [16]. The β2-AR subtype is a G protein-coupled receptor (GPCR); its stimulation causes the Gαs subunit mediated cyclic AMP (cAMP) synthesis and the consequent Protein Kinase A (PKA) phosphorylation. The PKA could be identified as one of the key effectors of the β2-signaling, together with the guanine nucleotide exchange protein (EPAC) subjected to the cAMP modulation. PKA elicits its effect by activating the PI3K/Akt/mTOR and Src/Ras/MAPK axis, while EPAC downstream regulates the B-Raf and MAP/extracellular signal-regulated kinases 1/2 (ERK1/2) through the Ras-related protein Rap-1A [17–19].

In head and neck squamous cell cancer (HNSCC), an epidermal growth factor receptor (EGFR) chimeric monoclonal antibody (mAb) (Cetuximab—CTX), is the standard of care of patients intractable with cisplatin, or in case of high recurrence and metastasis [20, 21]. The EGFR plays a key role in the pathogenesis and progression of HNSCC, being overexpressed in more than 80% of patients both in the tumor and in the surrounding tissue [22–24]. Its constitutive activation promotes cancer growth and progression mainly by the mitogen-activated protein kinase (MAPKs) and/or the PI3K/AKT/mTOR and JAK/STAT signaling [25]. Unfortunately, the therapy with CTX is often hindered by resistance mechanisms which make it ineffective. EGFR blockade often leads to oxidative stress increase in cancer cells, which, in turn, can drive cell death [26]. Nevertheless, cancer cells can boost their redox balancing machine and become resistant to this treatment [27].

In a previous study, in HNSCC, we have shown that the contemporary blockade of β2-AR and MEK1/2 has a synergistic effect that boosts cytotoxicity and autophagy and prevents resistance; in fact, β2-AR blockade drives a cell oxidative stress by the inhibition of the nuclear factor erythroid 2-related factor 2 (Nrf2). The latter regulates the expression of genes involved in oxidative stress response and drug detoxification [28]. Cells become resistant to chemical carcinogens and inflammatory stressors when NRF2 is activated.

Therefore, in this work, we have investigated the potential of the combined treatments with CTX and β2-AR inhibitors in an orthotopic model of HNSCC.

## Results

### Effects on UMSCC 103 viability of the Β2-AR inhibitor alone and in combination with U0126 and cetuximab

**Fig. 1:**
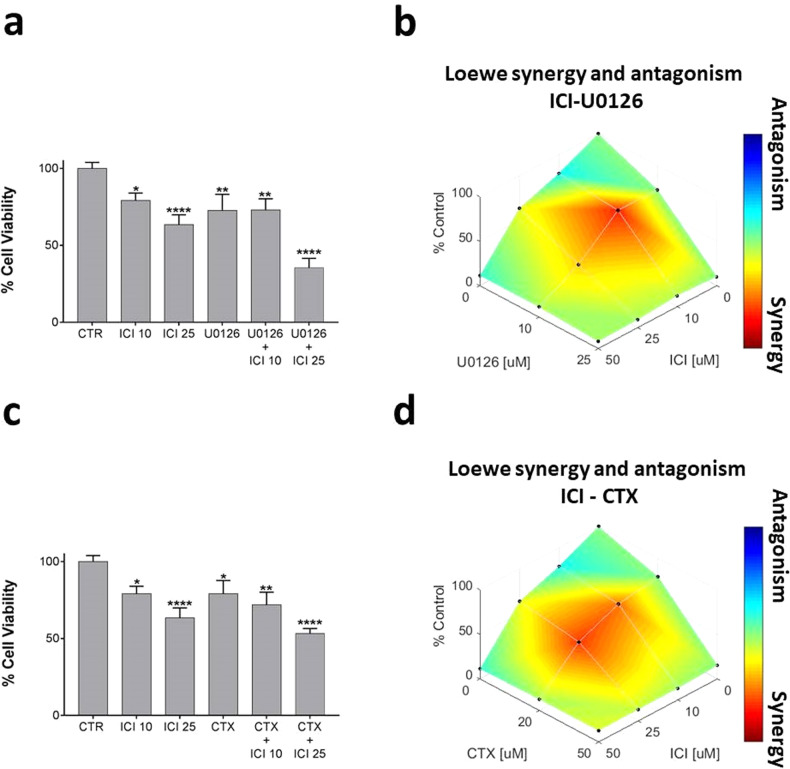
UMSCC 103 viability and synergism analysis after treatment with ICI, U0126, and CTX alone or in combination. **a** Viability assay on UMSCC 103 treated with ICI and U0126 at 48 h. The cell death was statistically significant after a single treatment with ICI (10 μM and 25 μM) and U0126 (10 μM); the cytotoxicity increases after treatment with a combination of drugs, with a dose-dependent relationship. **b** The viability coefficients related to the ICI/U0126 tested concentrations have been plotted in a combination index function to assess the drug’s synergism. The combination of drugs led to a cytotoxic synergic effect at 10 μM ICI + 10 μM U0126, which becomes milder at 25 μM ICI + 10 μM U0126. **c** Viability assay on UMSCC 103 treated with ICI and CTX at 48 h. The drug combination was very effective in a dose-dependent trend. **d** Synergism analysis of ICI and CTX cell death mediated, with significant results obtained with several combinations: 10 μM ICI + 20 μM CTX and μM ICI + 20 μM CTX (*P ≤ 0.05; **P ≤ 0.01; ****P ≤ 0.0001 vs. CTR).

According to our previous results, we verified the cytotoxic effect of the β2-AR inhibition in the UMSCC 103. As expected, cell viability was significantly reduced after 48 h of treatment with ICI (Fig. 1a), with a dose-dependent effect that finally reached 33% at 25 μM. Furthermore, the MEK 1/2 inhibition with U0126 also resulted in highly effective in promoting significant cytotoxicity at 10 μM. The ICI and U0126 synergism in UMSCC 103 has been statistically confirmed (Fig. 1b). Considering the strong EGFR upregulation in the UMSCC lines [29], and the adoption of Cetuximab (CTX) as the standard of care for the treatment of HNSCC [20, 21], we preliminarily tested the cytotoxic effect of this drug in UMSCC 103 (Fig. 1c). Effectively, we found a significant cell death at 20 μM, which hugely increased after co-treatment with CTX and ICI, in a dose-dependent manner. Even in this case, the drugs resulted synergic in promoting the UMSCC 103 cytotoxicity (Fig. 1d).

### Β2-AR and EGFR inhibition strongly delays the HNSCC progression in vivo

**Fig. 2:**
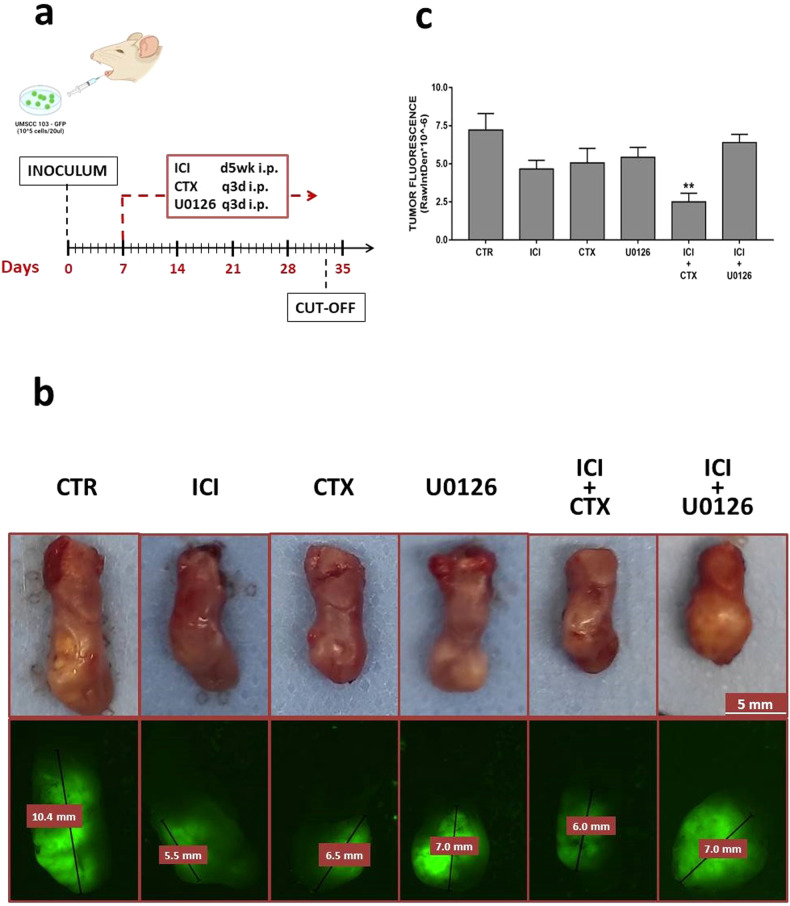
In vivo tumor growth assay. **a** Schematic in vivo experimental design. **b** Ex vivo tongue collection and tumor fluorescence documentation. **c** Tumor growth rate of mice treated with CTX and U0126 alone or in combination with ICI. A significant delay is reported in ICI + CTX-treated tumor-bearing mice (**P ≤ 0.01 vs. CTR).

We established an orthotopic HNSCC mouse model by injecting the UMSCCs 103^GFP^ directly into the nude mice tongues [30]. We have previously shown that this model replicates the characteristics of the original tumor and is a reliable model to study HNSCC. One week post injection we randomized the mice, after tumor fluorescence evaluation, and started the treatments with U0126, CTX, and ICI alone or in combination, which lasted almost four weeks; the mice were sacrificed once achieved the experimental cut-off (Fig. 2a). The UMSCC 103^GFP^ engraftment has been localized in the anterior part of the tongues. The tumors were very well localized into the distal portion of the tongue, with rare erythematous plaques and ulcers. The tumor histology highlighted a conventional type of squamous carcinoma, with the presence of parakeratotic cells fairly uniform and a basaloid appearance. These cells showed a distinctive pearl-like shape, with many intercellular connecting bridges made of keratin.

Nevertheless, the exponential cancer cell growth led to the invasion of most of the muscular organs (Fig. 2b), especially in the control group, where we observed also some ulcers. The tumor growth rate has been verified by monitoring the tumor mass fluorescence through the MacroFluo technology twice per week in mice under anesthesia with isoflurane 4%. During the follow-up, no significant loss of weight has been registered. Here we found a tumor growth delay (not statistically significant) after treatment with ICI at the concentration of 2 mg/Kg [31](≃35% fluorescence reduction). Interestingly, neither CTX (40 mg/kg) [32] nor U0126 (10 mg/kg) [33] alone were effective on the tumor growth, while we observed a significant tumor mass reduction (≃−65%) in mice subjected to the contemporary administration of ICI and CTX (Fig. 2C).

### Β2-AR expression pattern in HNSCC orthotopic mouse model

The β2-AR pathway is targeted by different selective and non-selective agonizts/antagonists for the treatment of several diseases. Few high doses or a series of small doses of these drugs in patients can induce the β-Arrestin mediated β2-AR desensitization, with the consequent tachyphylaxis [34]. These mechanisms are very well described in the case of the use of the β2-AR agonist, but little is known about the long-term effects of the β2-AR antagonists.

**Fig. 3:**
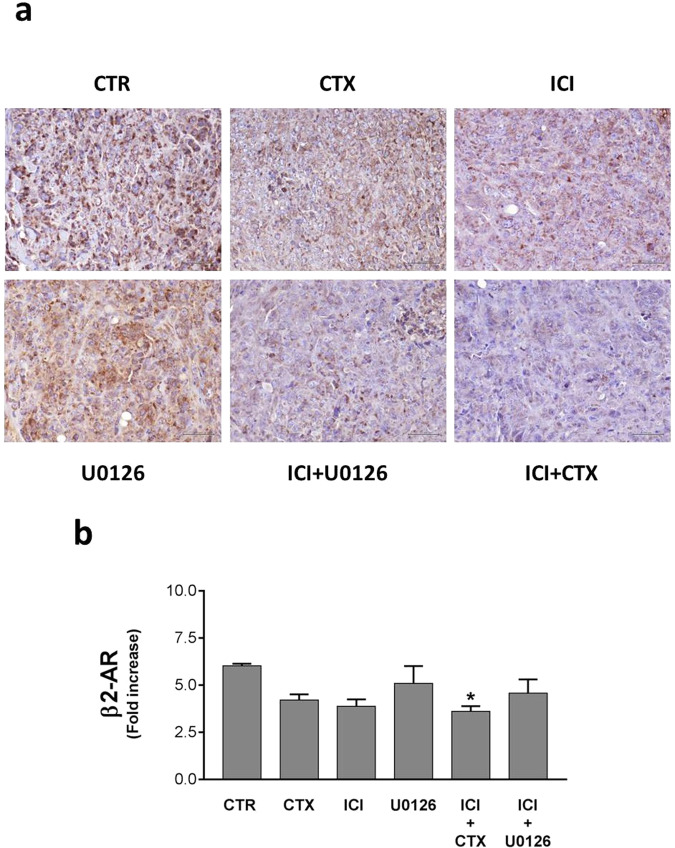
β2-AR expression in HNSCC mouse model. **a** Immunohistochemistry assay on mice tongues engrafted with UMSCC 103 for the β2-AR detection. This receptor was highly expressed in the untreated, while the CTX or ICI treatment induced its strong down-regulation. The inhibitory effect is more evident if we combine the drugs. **b** Western Blot analysis of the β2-AR modulation in cancer cells collected from the tumor bulk. The treatment of ICI and CTX significantly reduced the adrenoceptor expression (*P ≤ 0.05 vs. CTR).

In our HNSCC in vivo model, we analyzed the β2-AR modulation by immunohistochemistry (IHC), demonstrating a strong membrane expression of the β2-AR in the CTR group (Fig. 3a). Interestingly, although the intensity of the β2-AR signal and the percentage of positive cells were mildly reduced after treatment with CTX or U0126, in the ICI group we found a strong inhibition of β2-AR expression, which become noticeable after treatment with ICI in combination with both U0126 and CTX.

The β2-AR expression has been analyzed also by western blot (Fig. 3b). Here, we found that the β2-AR downregulation was statistically significant only in mice co-treated with ICI and CTX. Nevertheless, for the other groups, we observed the same trend described in the IHC (Fig. 3b; Fig. S1).

### Interplay between Β2-AR and EGFR/MAPK axis

**Fig. 4:**
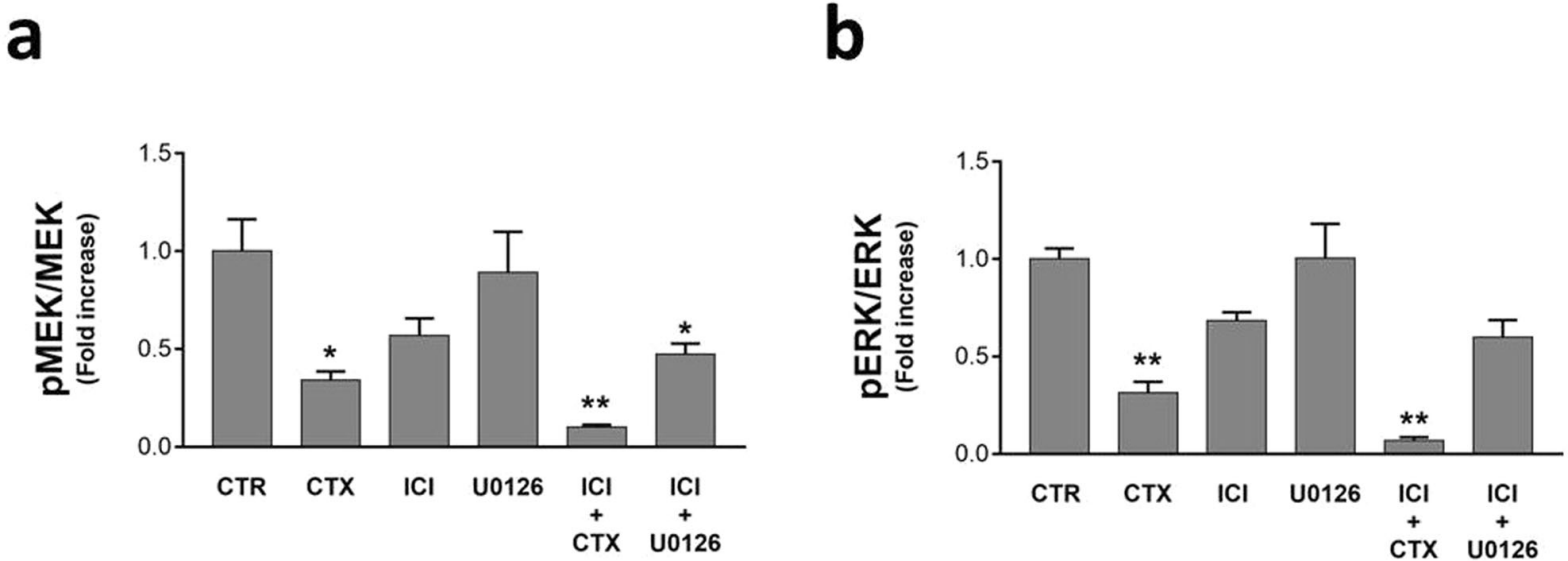
β2-AR and EGFR pathways crosstalk. Western Blot analysis, for assessing the activation levels of the MAPK pathway modulated by β2-AR and the master upstream regulator EGFR. **a** pMEK/MEK ratio is significantly affected by CTX alone, much more in combination with ICI. **b** The ERK phosphorylation status followed the same trend observed with pMEK/MEK. (*P ≤ 0.05; **P ≤ 0.01 vs. CTR).

Recent studies demonstrated the crosstalk between the β2-AR pathway and many other molecular mechanisms in cancer, among which the EGFR, which could be directly activated by the β2-AR [35]. Our previous results demonstrated the interplay between the β2-AR/cAMP/PKA and the MAPK/MEK/ERK axis in affecting the UMSCC 103 viability [28]. Surprisingly, in our in vivo model, we found that the selective not-competitive MEK inhibitor U0126 was not effective at all (Fig. 4a; Fig. S2), while CTX significantly blocked the MEK phosphorylation (≃−66%), even more when in combination with ICI (≃−90%). ICI single treatments promoted only a mild phospho-MEK down-regulation (≃−43%) which increased when this inhibitor was combined with U0126 (≃−53%). ERK phosphorylation was strongly blocked (≃−69%) by CTX and, remarkably, if in combination with ICI (≃−94%) (Fig. 4b; Fig. S2).

### UMSCC 103 ROS metabolism is modulated by the EGFR- Β2-AR axis crosstalk

The reactive oxygen species (ROS) play a fundamental role in the HNSCC development and drug resistance [36, 37]. Recent studies showed that EGFR constitutive activation dramatically impacts the ROS balance. In particular, the CTX promotes lipid peroxide accumulation due to the p38/Nrf-2/HO-1 axis impairment and the consequent cytotoxicity [38]. We know that the β2-AR pathway also plays a crucial role in the oxidative stress balance of the UMSCC 103 where we observed in vitro the Nrf-2 nuclear translocation inhibition after treatment with ICI, with the relative downregulation of the antioxidant enzymes involved in the ROS metabolic machinery [28].

**Fig. 5:**
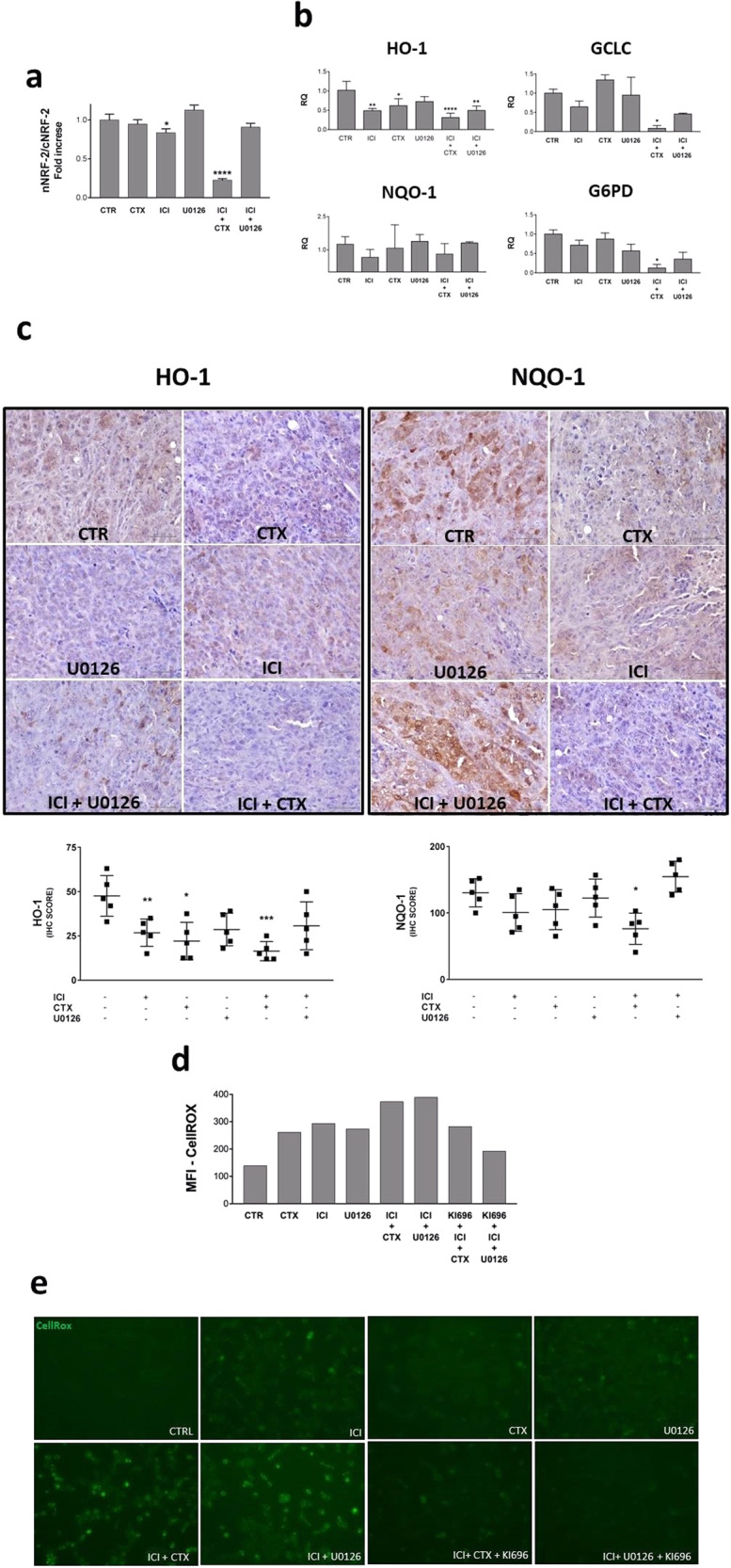
β2-AR and EGFR pathways regulate the ROS metabolism NRF-2 mediated. Evaluation of the ROS metabolism in cancer cells directly obtained from the HNSCC-bearing mice. **a** Western Blot analysis of Nrf-2 expression in both cytoplasmic and nuclear protein extracts. ICI treatment reduced the Nrf-2 nuclear translocation, in a more evident way if in combination with CTX. **b** RT-PCR for the HO-1, NQO-1, GCLC, and G6PD gene expression level analysis. The β2-AR blockade diminishes the gene expression level of HO-1, with a synergistic effect in combination with CTX, which is able to induce a milder effect by itself. The MEK 1/2 inhibition did not replicate this effect. No significant effects have been observed about the NQO-1 expression. The expression of GCLC and G6PDH is significantly reduced only after treatment with ICI in combination with CTX. **c** Immunohistochemistry assay on mice tongues engrafted with UMSCC 103 for the HO-1 and NQO1 detection. The ICI and CTX treatments sensibly reduced the expression of HO1, which increases if we combine the drugs. The expression of NQO1 was affected only in mice subjected to the combination of ICI plus CTX. **d** Cell-ROX assay for the evaluation of the oxidative stress in UMSCC 103 induced by our drugs. With both flow cytometer and **e** fluorescent microscopy analysis, we observed an increased level of oxidation after treatment with the β2-AR inhibitor, as well as with CTX and U0126. The same drugs are even more effective in combination, but this increased effect is counteracted by the KI696. (*P ≤ 0.05; **P ≤ 0.01; ****P ≤ 0.0001 vs. CTR).

We verified the level of Nrf-2 nuclear translocation in tumor cells (Fig. 5a; Fig. S3). As expected, we observed a strong Nrf-2 activation in the untreated group, with a high nuclear localization. On the other hand, in the ICI treatment group, Nrf2 was partially cytoplasmatic (≃−17%) and in mice co-treated with ICI and CTX it was almost completely cytoplasmatic (≃−78%).

Nrf-2 is the master regulator of many enzymes involved in ROS metabolism, among which are HO-1, Gclc, G6PD, and NQO-1. In our setting, we have found (Fig. 5b) that the ICI-mediated Nrf-2 cytoplasmic blocking significantly inhibits the HO-1 gene expression level (≃−52%). Quantitatively, we observed the same effect in mice co-treated with ICI and U0126. In the same way, CTX downregulated the HO-1 gene expression (≃−39%), and its effect is amplified when in combination with ICI (≃−69%). Gclc and G6PD gene expression are significantly reduced after co-treatment with ICI and CTX.

The NQO-1 gene expression analysis did not show significant results (Fig. 5b). We did not find a statistically validated variation of the NQO-1 gene expression in cancer cells extracted from mice tongues after the described treatments. In this case, we only observed a similar trend compared to the HO-1 data.

The IHC analysis (Fig. 5c) suggestively confirmed the HO-1 downregulation after treatment with ICI (IHC_score_ ≃ −44%). In addition, this inhibitory effect increases after co-treatment with ICI and CTX (IHC_score_ ≃ −66%). The contemporary treatment of EGFR and β2-AR inhibitors promoted the NQO-1 protein reduction (IHC_score_ ≃ −42%) (Fig. 5c).

To confirm that the effect on oxidative stress was mediated by the inhibitory effect on NRF2, we performed an in vitro experiment with a vital ROS stain (CellRox), combining our treatments with an NRF2 activator (KI696). Interestingly, we found that KI696 reduced ROS accumulation mediated by both ICI-CTX and ICI-U0126 combination (Fig. 5d, e)

## Discussion

The β2-AR pathway is involved in several biological mechanisms underlying the initiation and progression of many malignancies. Recent studies on ovarian cancer demonstrated that the β2-AR agonist stimulation accelerates tumor growth, similarly as it happens in patients undergoing chronic stress [11]. Also, in prostate and breast cancer, the β2-AR activation promotes the cancer cell survival through the inhibition of the pro-apoptotic mediators [10]. Makoto et al. first described in vivo how the stimulation of the β2-AR induces DNA damage by suppressing p53 via the β-arrestin/AKT/MDM axis [39]. Conversely, the β2-AR antagonists can delay the tumor growth or eventually attenuate its metastatic potential, for example, suppressing the secretion of the matrix metalloproteinase (MMPs) in several tumors, affecting their invasive potential [40].

Our previous study highlighted the synergistic interplay between the β2-AR and MAPK pathways in HNSCC. In particular, the contemporary inhibition of β2-AR and MEK1/2 promoted the UMSCC 103 cell death by the down-regulation of the PI3K/Akt/mTOR, p38, and NFkB pathways and the contemporary Nrf-2 blocking [28]. Nilsson et al. described the involvement of the β2-AR activated pathway in the EGFR TKI resistance due to a LKB1/CREB//IL-6-dependent mechanism [41]. Comparable results have been achieved in a retrospective clinical study where the Her2-overexpressing metastatic breast cancer patients, subjected to the concurrent treatment with β-blocker propranolol and trastuzumab, showed an improvement of progression-free survival and overall survival, underling the strong interplay between the β2-AR and EGFR pathways [16]. Similarly, in HNSCC patients, there is a correlation between β2-AR expression and poor prognosis [42]. Based on these data, we decided to investigate the role of the β2-AR pathway in an HNSCC mouse model, focusing on the interplay between the above-described pathways. Therefore, we initially showed in vitro that the inhibition of β2-AR and MAPK is synergic in reducing the viability of UMSCC 103. Subsequently, considering that MEK 1/2 is a downstream EGFR pathway component, very often upregulated in resistance to therapies tumors [43], we decided to introduce the CTX in our study, widely considered as the standard of care of many HNSCC patients [44]. Effectively, the CTX was significantly cytotoxic on UMSCC 103 and its efficacy increases in combination with ICI, in a synergistic way.

In many types of β2-AR expressing cells, the prolonged and continuous agonist stimulation leads to an initial acute cAMP production which slowly declines almost to the basal level. This mechanism belongs to the tachyphylaxis process, characterized by the inactivation of the G-protein signaling and the contemporary cytoplasmic internalization of the receptor, balanced by the βARK activation [45, 46]. Recent studies demonstrated that in normoxia, the β2-blockers sensibly promote the phosphorylation of the intracellular receptor domains needed for the βARK-mediated endosome internalization and the consequent receptor desensitization. In hypoxia conditions, this mechanism is completely reverted; in fact, the receptor-expressing balance is turned to a strong re-sensitization [47]. Consistently with these findings, in untreated mice, we found a high β2-AR expression which was differently modulated in the case of long-term treatments with both β-blockers and EGFR inhibitors. In mice with bigger and more necrotic tumor masses, we observed a strong β2-AR cell membrane expression, which is strongly inhibited by the long-term treatment with the β2 selective antagonist ICI, and even more when in combination with CTX. In our study, the down-regulated expression of the β2-AR correlates with smaller tumor masses.

To further understand the mechanisms underlying the cytotoxicity of the β2-AR and EGFR inhibition, we investigated the pathways regulated by these receptors. Here we found a significant impairment in the MAPK signaling due to the blockade of the MEK phosphorylation by both ICI and CTX. Surprisingly, although U0126 was a selective MEK 1/2 inhibitor, it was not effective, probably because of the pharmaceutical limitations that compromised its utility as an in vivo anticancer agent [48]. For this purpose, we studied in deep the ROS metabolism machinery, finding the impairment of the Nrf-2 signaling in our HNSCC mouse model. We know that in basal condition, Nrf-2 is sequestered into the cytoplasm by its cytoplasmic chaperone molecule Kelch‐like‐ECH‐associated protein 1 (Keap1). The cell exposition to xenobiotic and oxidative stress reduces the affinity of Keap-1 with Nrf-2, which is released to the nucleus where it trans-activates the antioxidant responsive elements (ARE) sequence, leading to the synthesis of several proteins such as the xenobiotic detoxification enzyme NQO-1, the catalytic subunit in rate‐limiting step of GSH synthesis GCLC, the first pentose phosphate pathway enzyme G6PD and the Heme metabolism enzyme HO-1 [49]. Also, in oral pre-cancerous leukoplakia or erythroplakia yet, there are high ROS levels, due to the activation of several oxidative enzymes, such as the inducible nitric oxide synthase (iNOS), with the relatively augmented risk of DNA damage in oral epithelium [36]. Interestingly, in our model, we found a strong activation of the ROS metabolism due to the Nrf-2 nuclear translocation. Conversely, the treatment with ICI significantly blocked the Nrf-2 into the cytoplasm, with the relative down-regulation of the genes involved in the ROS machinery. Surprisingly, also CTX was highly effective in reducing the Nrf-2 activity, as already described by some authors. Indeed, recent studies in NSCLC patients described the activation of the Nrf-2 mediated ROS metabolism by the EGFR blockade [50] or by the presence of a dysfunctional Keap1. In colon cancer patients treated with CTX has been observed an enhanced RSL3 ferroptosis by inhibiting the Nrf-2/HO-1 signaling [38]. Interestingly, CTX and ICI, together, were more able to reduce the Nrf-2 translocation; in the same way we have described this behavior for the MEK/ERK signaling. Effectively, the MAPK signaling is strictly correlated with the ROS metabolisms in several cancers [51, 52], among which the HNSCC [28]. Moreover, we demonstrated that ROS accumulation is correlated to Nrf-2 modulation by ICI-CTX and ICI-U0126 combination. In fact, using KI696 (an Nrf-2 activator), the effect of this combination on ROS accumulation was strongly reduced. Our data mechanistically suggested the correlation between the inhibition of the ERK/MEK pathway, by the contemporary inhibition of the β2-AR and EGFR pathways, and the Nrf-2 activation, with the contemporary blockade of the downstream ROS metabolism enzymes HO-1, GCLC, G6PD, and NQO-1.

Further studies are needed to understand the interplay between the EGFR selective inhibition and the β2-AR desensitization balance.

## Conclusion

In conclusion, our findings suggest a powerful interplay between the β2-AR and EGFR signaling in HNSCC. In particular, the contemporary inhibition of these pathways significantly reduces the tumor growth in the orthotopic mouse model of HNSCC due to the impairment of the MEK/ERK/Nrf-2 axis. Indeed, we found a strong inhibition of the ERK phosphorylation by ICI and CTX directly into the tumor sections or in ex vivo collected tumor cells, showing a synergistic effect when administered in combination. We have also demonstrated that the MAPK signaling impairment affects the Nrf-2-regulated ROS metabolism. In fact, we have observed that the Nrf-2 cytoplasm blockade induced by the treatments with the relative down-regulation of HO-1 and NQO-1 enzymes leads to an enhanced cytotoxicity due to the ROS unbalancing.

Our findings, taken together, suggest that in HNSCC, the inhibition of both β2-AR and EGFR signaling is a potential target to strongly reduce tumor cell growth.

## Materials and methods

### Chemicals, cell culture, and in vitro treatment

All chemicals were purchased from Sigma-Aldrich (St. Louis, USA) unless otherwise specified. Selective inhibitors of β2-AR (ICI118,551) and MEK1/2 (U0126) were obtained from Tocris Bioscience (Bristol, United Kingdom). Cetuximab was obtained from Merck KGaA (Merck KGaA, Darmstadt, Germany). KI696 was obtained from (MedChemExpress, Monmouth Junction, USA)

UMSCC103-GFP (engineered HNSCC cell line) used in this study was established at the University of Michigan under a protocol approved by the Institutional Review Board Office under the university’s regulations and described here. The human embryo kidney cell line (HEK 293 T) was obtained from the American Type Culture Collection (ATCC, Manassas, VA). Cells were cultured in DMEM (Gibco, NY, USA) supplemented with 2 mM glutamine, 100 IU/mL penicillin, 100 μg/mL streptomycin (Invitrogen, Carlsbad, CA), and 10% heat-inactivated fetal bovine serum (FBS) (Gibco, NY, USA) at 37 °C in a humidified atmosphere under 5% CO_2_. The cell line was kept mycoplasma-free; checking was performed every three months.

### Establishment of UMSCC103-GFP

The UMSCC103-GFP has been obtained with the gene editing technology, based on the use of the pLenti CMV GFP Puro (658-5) (a gift from Eric Campeau & Paul Kaufman) (Addgene plasmid # 17448; http://n2t.net/addgene:17448; RRID: Addgene_17448) lentiviral vectors [53]. The day before the transfection, 5 × 10^6^ of HEK293T cells were seeded in a 10 cm dish. Transfection was done with 50 µl of Lipofectamine 2000 (Invitrogen) according to the manufacturer’s instructions using 15 µg of the transfer vector, 15 µg of pLP1 (Invitrogen), 6 µg of pLP2 (Invitrogen), and 3 µg of pVSV-G (Invitrogen). At 48 and 72 h post-transfection, viral supernatants were collected and filtered through a 0.2 µm syringe filter. Subsequently, the UMSCCs 103 have been transduced for 24 h at an MOI between 0.5 and 1, with 8 µg/ml of polybrene (Sigma). The fluorescent cells have been purified by FACS Aria III cell sorter (Becton & Dickinson, Mountain View, CA, USA).

### Cell viability assay

Cell viability was measured by the colorimetric 3-(4,5-dimethyl-2-thiazolyl)-2,5-diphenyltetrazolium bromide (MTT) assay. Cells were seeded in 96-well plates at a density of 10^4^ cells per well, then they were treated with 100 μL of 1 mg/mL MTT (Sigma) in DMEM medium containing 10% fetal bovine serum for 4 h at 37 °C. The medium was then replaced with 200 μL of DMSO and shaken for 15 min, then absorbance at 540 nm was measured using a microplate ELISA reader with DMSO used as the blank. To quantify the synergistic or antagonist effect of the drug combinations, Combenefit® software was used [54]. Each sample was performed in triplicate.

### In vivo studies

HNSCC mouse models have been carried out on 8-week-old female Athymic Nude-Foxn^ŋu^ nu/nu mice from Envigo ***(Envigo RMS Srl*** *S*. *Pietro al Natisone—Udine Italy*). Mice were housed in a group of seven in a 12 h light: 12 h dark cycle in a controlled room temperature of 22 ± 2 °C and fed ad libitum. For the xenograft orthotopic HNSCC model, the mice have been previously anesthetized by intra-peritoneal injection of a solution of Zoletil 100, 50 mg/kg (Virbac), according to their body weight. Subsequently, the Xenograft orthotopic mouse model of HNSCC has been generated by injecting UMSCC103-GFP (10^5^ in 50 µl of PBS) directly into the anterior part of the tongue. The tumor growth has been evaluated in mice under isoflurane anesthesia, with the Macrofluo microscope (Leica; Wetzlar, Germany) documentation system. The tumor pictures were analyzed with the ImageJ software to evaluate the fluorescence intensity. After reaching the fluorescence value of ~10^6^ RawIntDen, the mice (*n* = 5) were equally divided into six groups based on different treatments:

1. CTR group: 4 weeks of treatment with normal saline solution;
2. ICI118,551 (ICI) groups: 4 weeks of treatment (2 mg/kg 5 days per week for 4 weeks, intraperitoneally).
3. Cetuximab groups: 4 weeks of treatment (40 mg/kg every 3 days, intraperitoneally);
4. U0126 groups: 4 weeks of treatment (10 mg/kg every 3 days, intraperitoneally).

In the same way, we monitored the mice during the follow-up, collecting the tumor fluorescence data twice per week until they reached the ethical endpoint.

This study was approved by the Italian Animal Ethics Committee of “Istituto Nazionale deiTumori Fondazione G. Pascale”, Naples, Italy. All the experiments were performed by also following the European Directive 63/2010/UE and the Italian Law (DL 26/2014, authorized by the Ministry of Health, prot. #647/2020-PR Italy). This study was carried out in accordance with the recommendations that cover all scientific procedures involving the use of live animals, as we have previously reported.

### Immunohistochemistry

Immunohistochemical staining was carried out on tumor whole slides to evaluate the expression of HO1, NQO1, and ß2. Paraffin slides of 0.4 μm thickness were analyzed using the following antibodies: anti-HO1 (rat monoclonal, R&D SYSTEM cat. N. MAB3776) (diluted: 1:100) anti-NQO1 (mouse monoclonal, R&D SYSTEM cat. N. MAB7567) (diluted: 1:100) and anti-ß2 (rabbit polyclonal, Santa Cruz, cat. N. sc-569) (diluted: 1:100).

IHC was performed using BOND Polymer Refine Detection (Leica Biosystem, Milan, Italy) as a fully automated assay on the BOND RX (Leica Biosystems), according to the manufacturer’s instructions. The BOND Polymer Refine Detection kit contains a peroxide block, post primary, polymer reagent, 3,3′-diaminobenizidine tetrahydrochloride hydrate chromogen, and hematoxylin counterstain. Expressions of the biomarkers were evaluated semi-quantitatively based on the staining intensity and the number of immunoreactive cells. The immunohistochemical staining was scored as follows: no staining or weak staining in <10% of tumor cells, score 0; weak staining in >10% of tumor cells, score 1+; moderate staining in >10% of tumor cells, score 2+; strong staining in >10% of tumor cells, score 3+.

### Immunoblot analysis

Cells were lysed in 2% SDS containing 2 mM phenyl-methyl sulphonyl fluoride (PMSF) (Sigma), 10 μg/ml antipain, leupeptin and trypsin inhibitor, 10 mM sodium fluoride and 1 mM sodium orthovanadate (all from Sigma) and sonicated for 30 s. Proteins of whole-cell lysates were assessed using the Lowry method, and equal amounts were separated on SDS-PAGE. The proteins were transferred to a nitrocellulose membrane (Schleicher and Schuell, BioScience GmbH, Germany) by electroblotting. The balance of total protein levels was confirmed by staining the membranes with Ponceau S (Sigma). Immunoblotting was performed with the following antibodies: anti-β2-AR (H-20), anti-ERK2 (C-14, positive also for ERK1), anti-phospho-ERKs (E-4), anti-MEK 1/2 (9G3), anti-phospho MEK 1/2 (7E10), anti-GAPDH (6C5), Anti-β-actin (C4), and anti-Histone H3 (1G1) all from Santa Cruz Biotechnology (Santa Cruz, CA); anti-NRF2 (from Invitrogen #PA5-88084, Waltham, Massachusetts, USA). Peroxidase-conjugate anti-mouse or anti-rabbit IgG (Amersham-Pharmacia Biotech, UK, or Santa Cruz) were used for enhanced chemiluminescence (ECL) detection. Each western blot was performed in triplicate.

### Cellrox assay

Cells were plated on glass-bottom 35-mm MatTek dishes and treated with ICI and/or U0126- CETUXIMAB for 24 h and 1 μM KI696 for 24 h at 37 °C. The cells were then stained with 5 μM CellROX green reagent by adding the probe to the complete media and incubating at 37 °C for 30 min. The cells were then washed with PBS and then imaged on a fluorescence microscope EVOS M5000 Imaging System (Thermo Scientific, Rockford, USA). For flow cytometry, cells were detached and analyzed with a FACS CANTO II (BD Biosciences, San Jose, CA). Data were analyzed by FlowJo V10 software (FlowJo LLC, USA).

### RNA isolation and qRT-PCR

Total RNA was isolated by RNeasy Mini Kit (Qiagen) according to the manufacturer’s instructions; RNA was treated with DNase (Promega, Milan, Italy) to exclude DNA contamination and 1 μg total RNA reverse-transcribed using VILO SuperScript (Invitrogen, Monza, Italy). Gene expression assays were performed on a StepOne Thermocycler (Applied Biosystems, Monza, Italy), and the amplifications were carried out using SYBR Green PCR Master Mix (Applied Biosystems, Monza, Italy). The reaction conditions were as follows: 95 °C for 15 min, followed by 40 cycles of three steps consisting of denaturation at 94 °C for 15 s, primer annealing at 60 °C for 30 s, and primer extension at 72 °C for 30 s. A melting curve analysis was performed from 70 °C to 95 °C in 0.3 °C intervals. Each sample was performed in triplicate. Glyceraldehyde 3-phosphate dehydrogenase (GAPDH) was used to normalize for differences in RNA input. Primer sequences are reported in Table 1.

**Table 1:** Sequences of primers used.

G6PD	Forward Primer	CTGTTCCGTGAGGACCAGATCT
	Reverse Primer	TGAAGGTGAGGATAACGCAGGC
Gclc	Forward Primer	GGAAGTGGATGTGGACACCAGA
	Reverse Primer	GCTTGTAGTCAGGATGGTTTGCG
Nqo2	Forward Primer	GTATGCCATGAACCTTGAGCCG
	Reverse Primer	GCTCATCAGTGATGTCGCTAGC
Ho-1	Forward Primer	CCAGGCAGAGAATGCTGAGTTC
	Reverse Primer	AAGACTGGGCTCTCCTTGTTGC
GAPDH	Forward Primer	GTCTCCTCTGACTTCAACAGCG
	Reverse Primer	ACCACCCTGTTGCTGTAGCCAA

### Statistical analysis

Group differences were analyzed with a two-sided paired or unpaired Student’s *t*-test. In vivo experiments were repeated twice. Differences between groups analyzed with the *t*-test, Wilcox, or Mann–Withney were considered statistically significant for *p* < 0.05. Statistical analyses were performed with GraphPad Prism 7 software. Sample sizes were chosen based on preliminary results to ensure a power of 80% and an alpha level of 5%. No data or animals were excluded from the analyses.

## Supplementary information

Supplemental material

Reproducibility Checklist
