# Supplementary material for: β2-AR inhibition enhances EGFR antibody efficacy hampering the oxidative stress response machinery
Source: Cell Death Dis. 2023 Sep 19;14(9):613. doi: 10.1038/s41419-023-06129-9 (PMC10507049; doi:10.1038/s41419-023-06129-9)

**β_2_-AR inhibition enhances EGFR antibody efficacy hampering the oxidative stress response machinery**

Vitale Del Vecchio^#1^, Luigi Mele^#1^, Sameer Kumar Panda^1^, Ibone Rubio Sanchez-Pajares^1^, Laura Mosca^2^, Virginia Tirino^1^, Massimiliano Barbieri^3^, Francesca Bruzzese^3^, Federica Zito Marino^4^, Marina Accardo^4^, Giovanni Francesco Nicoletti^5^, Gianpaolo Papaccio^1*^, Antonio Barbieri^†3^, Vincenzo Desiderio ^†1*^

^1^ Department of Experimental Medicine, University of Campania "Luigi Vanvitelli", Naples, Italy

^2^Department of Precision Medicine, University of Campania "Luigi Vanvitelli", Naples, Italy

^3^Animal Facility, Istituto Nazionale Tumori, Istituto Di Ricovero e Cura a Carattere Scientifico "Fondazione G. Pascale", Naples, Italy.

^4^Department of Mental and Physical Health and Preventive Medicine, University of Campania "Luigi Vanvitelli", Naples, Italy

^5^Multidisciplinary Department of Medical-Surgical and Dental Specialties, University of Campania "L. Vanvitelli", Via L. de Crecchio 6, 80138 Naples, Italy.

**SUPPLEMENTARY MATERIAL**

**Fig.S1** Western blot assay for the detection of the β2-AR (original filter)


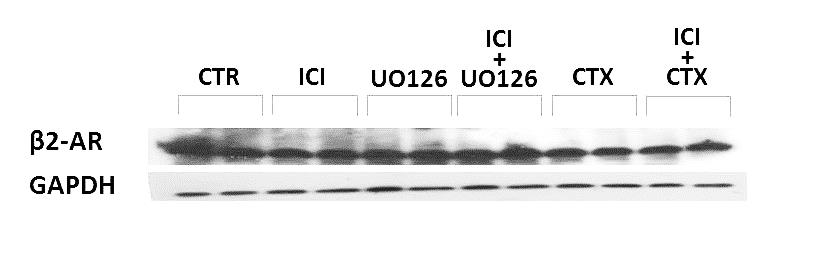


**Fig.S2** Western blot assay for the detection of the MEK, pMEK, ERK, pERK (original filter)


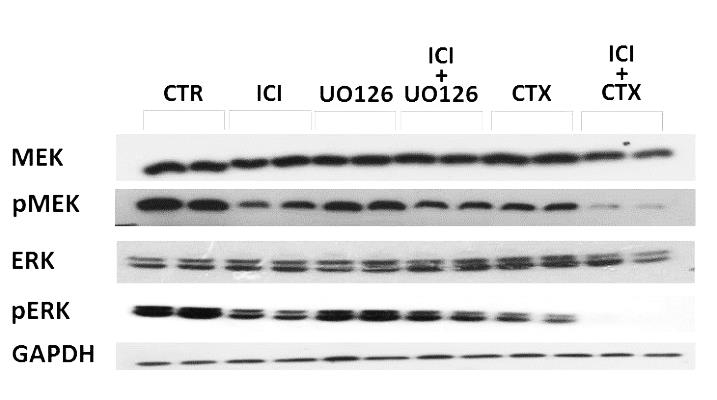


**Fig.S3** Western blot assay for the detection of the NRF-2 (original filter)


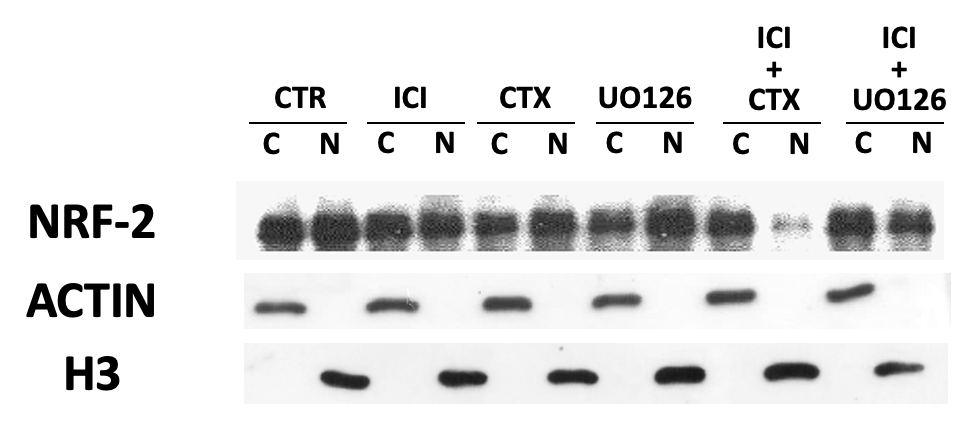


**Fig.S4** CellROX assay. Flow cytometer analysis of the oxidative stress.
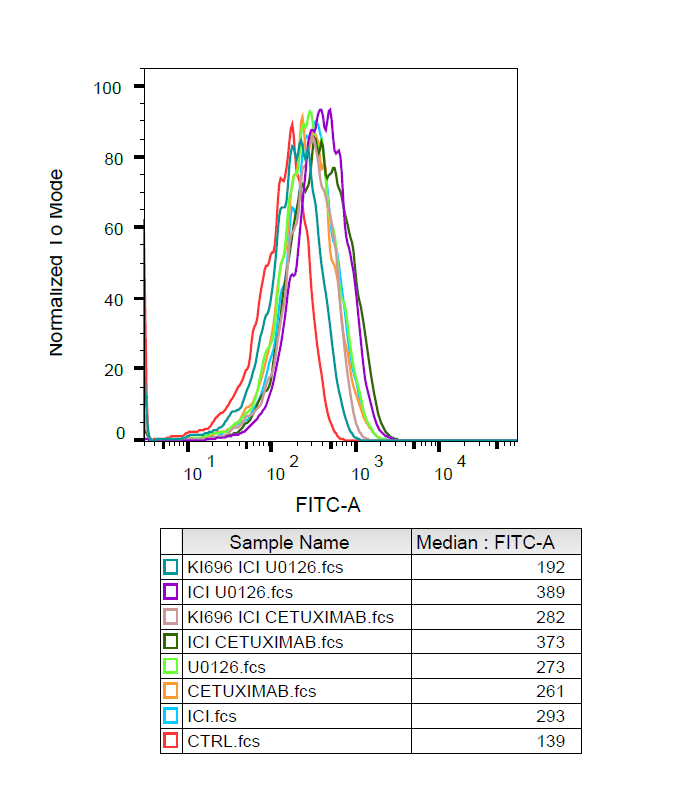

Supplement: Supplementary file 1 — Supplemental material [file 41419_2023_6129_MOESM1_ESM.docx]
